# First person – Yoshikazu Haramoto

**DOI:** 10.1242/bio.058759

**Published:** 2021-04-29

## Abstract

First Person is a series of interviews with the first authors of a selection of papers published in Biology Open, helping early-career researchers promote themselves alongside their papers. Yoshikazu Haramoto is first author on ‘Visualization of X chromosome reactivation in mouse primordial germ cells in vivo’, published in BiO. Yoshikazu is a Senior Researcher in the lab of Research Group Leader, TATENO Hiroaki at the Multicellular System Regulation Research Group, Cellular and Molecular Biotechnology Research Institute, National Institute of Advanced Industrial Science and Technology (AIST), Higashi, Tsukuba, Ibaraki, Japan, investigating mechanisms of development and regeneration.


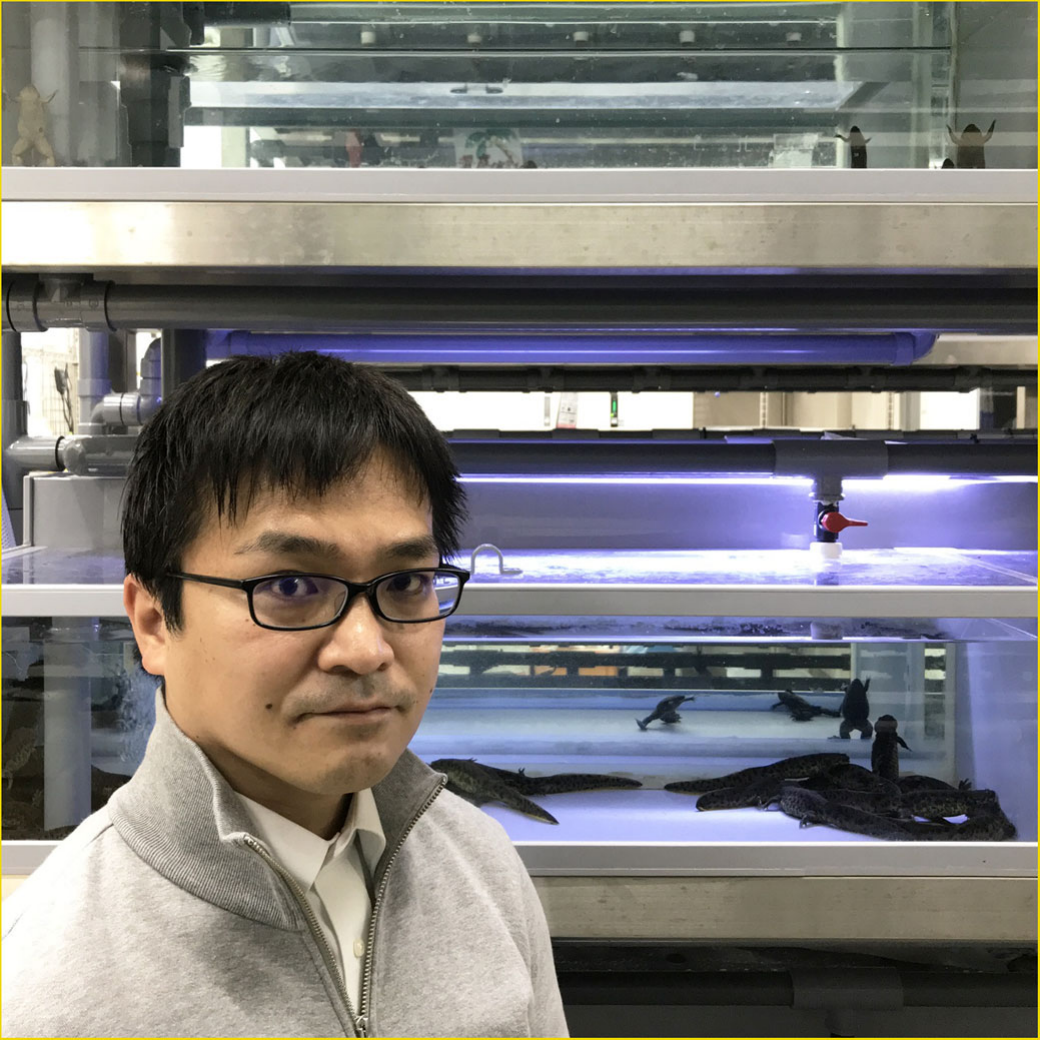


**Yoshikazu Haramoto**

**What is your scientific background and the general focus of your lab?**

I have been studying developmental biology, primarily in amphibians. My research is aimed at elucidating the mechanisms of organ and tissue formation. I am exploring applications for regenerative medicine, through research on the tissue regeneration mechanism of amphibians with high regenerative ability and stem cells of mammals. In mammals, the activated state of the X chromosome is understood as one of the indicators of stemcellness. In analyzing the properties of stem cells that contribute to tissue regeneration, I was interested in the mechanism of epigenetic changes in X chromosome inactivation and reactivation. In this study, we chose mouse primordial germ cells, which are known to undergo X chromosome reactivation *in vivo*, for our analysis. For me, this research was a new challenge and an exciting experience, such as changing the biological phenomena and animal species to be analyzed.

In addition to such basic research on developmental biology and cell biology, our research group is developing glycan analytical technology and quality-control technology for human stem cells, focusing on sugar chain profiling. The National Institute of Advanced Industrial Science and Technology (AIST) in Japan attracts many researchers with various research backgrounds. This makes it an attractive environment for our young researchers who wish to pursue interdisciplinary research.

**How would you explain the main findings of your paper to non-scientific family and friends?**

In mammals, sex is generally determined by whether an individual inherits two X chromosomes or one X and one Y. The Y chromosome contains extremely fewer genes than the X chromosome. Therefore, the expression of the X chromosome genes must be aligned between males, which have only one X chromosome, and females, which have two X chromosomes. In almost all cells in the female mouse, gene expression is regulated to the same level as in male mice by inactivating either the paternal or maternal X chromosome. The exception to this is primordial germ cells, in which both X chromosomes are active and the cells exhibit the ability to differentiate into any type of cells in the future. In this study, we have clarified when and where X chromosomes become activated in primordial germ cells. This research will also help to elucidate the mechanism by which cells acquire the ability to differentiate into all kinds of tissues.

“This research will also help to elucidate the mechanism by which cells acquire the ability to differentiate into all kinds of tissues.”

**What are the potential implications of these results for your field of research?**

In the fetal body, somatic-germ cell interactions are thought to be important for germ cell maturation, but their effects and mechanisms on reprogramming have not yet been elucidated. Our results indicated that environment of genital ridge is important for X chromosome reactivation and proliferation of germ cells. Elucidation of the developmental mechanism of germ cells is expected to contribute to the establishment of reconstituted egg production technology *in vitro* in the future.

**What has surprised you the most while conducting your research?**

It is interesting that there is no specific site inside the genital ridge that induces the reactivation of the X chromosome, and that it occurs uniformly inside that. It is also noteworthy that the reactivation of chromosomes progresses with the explosive proliferation of germ cells, suggesting the involvement of an active demethylation mechanism.

“… the reactivation of chromosomes progresses with the explosive proliferation of germ cells…”

**What, in your opinion, are some of the greatest achievements in your field and how has this influenced your research?**

Development of Momiji mouse system by Dr. Shin Kobayashi, my co-worker and the corresponding author of present paper had a great influence on my research (Kobayashi et al., 2016). Since X-chromosome reactivation is an indicator of cell pluripotency, the Momiji system, which can monitor X-chromosome activation state, is very attractive for stem-cell research. In this study, we used this system to visualize the activity of the X chromosome and succeeded in detecting reprogramming that occurs during germ cell development.

**Figure:**
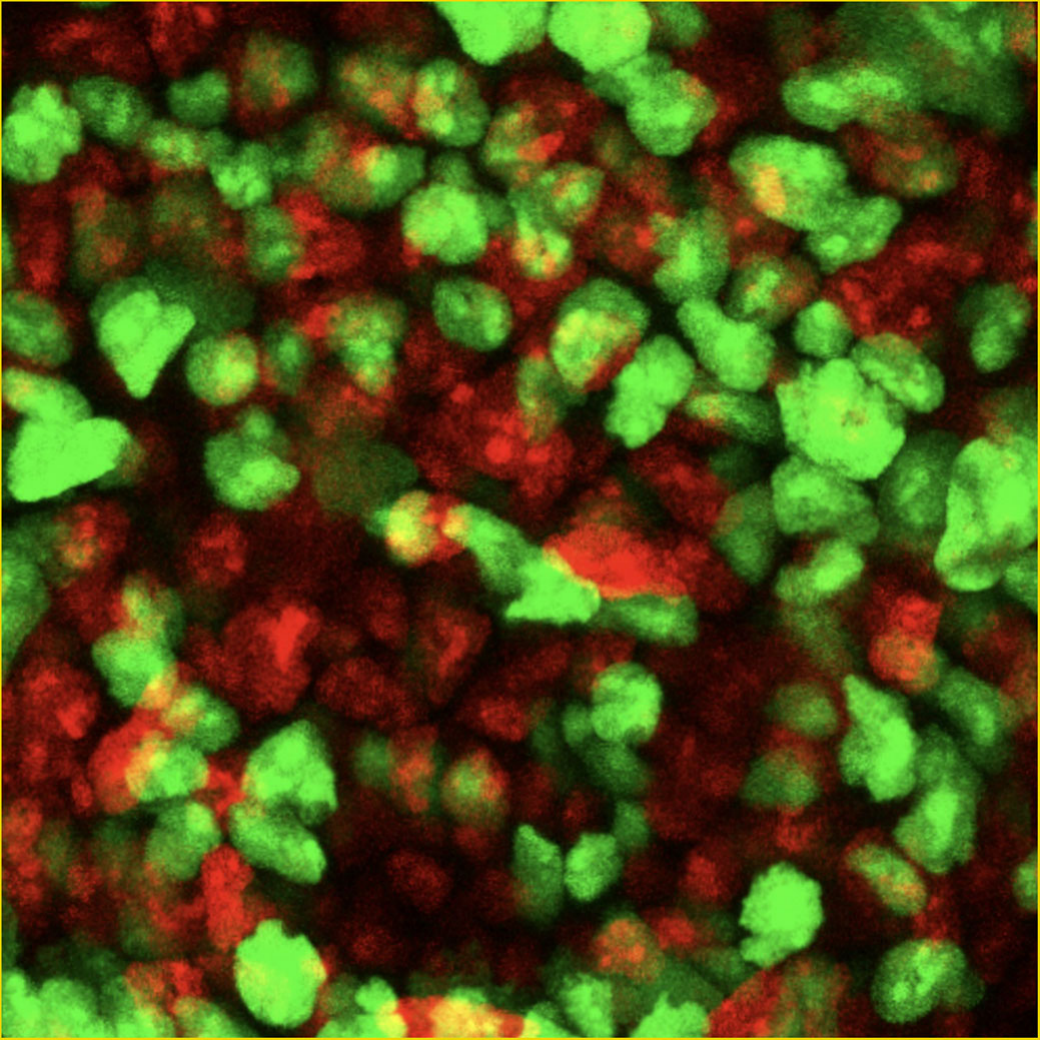
**The epiblast cells of Momiji mouse at E6.5.**

**What changes do you think could improve the professional lives of early-career scientists?**

Even if you don't immediately know what the discovery will do, it could be the foundation for changing the world in the future. Demanding the presentation of an indulgence that says ‘useful’ creates an atmosphere that forces everyone to come up with an easy-to-understand theme, which may hinder the curiosity and challenging spirit of young people. It is very important for the scientist to be able to share with society the value of knowing facts that have never been known before. More grant opportunities are needed for young scientists to pursue their research with creative ideas. Our institute has an environment that promotes internal communication between researchers. I would like to continue challenging with many scientists to gain insight into nature.

**What's next for you?**

The currently found X-chromosome reactivated cells are extremely limited in type and have pluripotent properties in common. Based on previous studies, X chromosome reactivated cells are expected to be absent or present in very small numbers elsewhere, making systematic search for X chromosome reactivation difficult using conventional detection methods. The Momiji mouse system can be a useful tool to solve this difficult problem. We would like to use this system to detect ‘X chromosome reactivation’ that occurs in the body. Since ‘X-chromosome reactivation’ can be used as an indicator to evaluate the undifferentiated nature of stem cells, I would like to explore new undifferentiated cells, and analyze and elucidate biological phenomena that are expected to involve reprogramming, such as tissue regeneration and the initiation of cancer formation.
